# Mechanisms by which proline reductase of *Clostridioides difficile* promotes efficient metabolism and disease progression *in vivo*

**DOI:** 10.1128/mbio.01180-26

**Published:** 2026-06-22

**Authors:** Laura M. Cersosimo, Madeline Graham, Auriane Monestier, Aidan Pavao, Jay N. Worley, Johann Peltier, Bruno Dupuy, Lynn Bry

**Affiliations:** 1 Massachusetts Host-Microbiome Center, Department Pathology, Brigham & Women's Hospital, Harvard Medical School. Boston 207095 , Boston, Massachusetts, USA; 2 Pathogenesis of Bacterial Anaerobes, Department of Microbiology, Institut Pasteur, Université Paris-Cité,UMR-CNRS 555089 , Paris, France; 3 Institute for Integrative Biology of the Cell (I2BC), Université Paris-Saclay, CEA, CNRS, 27048, Gif-sur-Yvette, France; 4 National Center for Biotechnology Information, National Library of Medicine, National Institutes of Health 2511, Bethesda, Maryland, USA; Universiteit Gent, Gent, Belgium

**Keywords:** commensal bacteria, Stickland amino acids, *Clostridioides difficile*, prdB, proline metabolism

## Abstract

*Clostridioides difficile* is a toxin-producing pathogen that opportunistically infects those with a depleted gut microbiome, often triggered by antibiotic use. *C. difficile* preferentially utilizes Stickland amino acids, including proline, to promote energy generation and growth for colonization. We evaluated host outcomes from infection with wild-type and Δ*prdB* mouse-infective *C. difficile* ATCC 43255 strains to investigate how proline metabolism modulates *C. difficile’s* pathogenesis and interactions with commensals in an enriched gut nutrient environment. While gnotobiotic mice infected with the Δ*prdB* mutant succumbed to infection, they showed delayed colonization and toxin production, thereby extending their survival. *In vivo C. difficile* transcriptomic analyses demonstrated a shift from Stickland fermentation to carbohydrate metabolism in the Δ*prdB* mutant. To investigate functions of *C. difficile’s* proline reductase in interactions with the commensal microbiota, we evaluated infection outcomes in mice co-colonized with the disease-promoting commensal *Clostridium sardiniense* (CSAR). One-third of CSAR and Δ*prdB-*infected mice survived, while co-colonized mice with the wild-type strain rapidly succumbed within 48 h. *In vivo* transcriptomic analyses from co-colonized mice identified additional metabolic defects in the Δ*prdB* mutant, including failure to express oxidative Stickland pathways and ornithine fermentations to harness CSAR-produced ornithine, suggesting conversion of the mutant into a glycolytic strain that was now in direct competition with CSAR for carbohydrate substrates. CSAR remained metabolically active during the Δ*prdB* infection through its mucin degradation and polysaccharide and disaccharide metabolism. Our findings illustrate critical functions of *C. difficile’s* proline reductase in coordinating early metabolism to facilitate gut colonization, including interactions with a cross-feeding commensal species.

**IMPORTANCE:**

*Clostridioides difficile* is a spore-forming bacterium that commonly causes pseudomembranous colitis in patients exposed to antibiotics, with infections leading to 30,000 deaths annually in the United States. We conducted further *in vitro* and *in vivo* analyses to determine how proline reductase modulates *C. difficile* colonization, growth, and metabolism, and showed that this pathway is critical to *Clostridium sardiniense’*s ability to cross-feed with the pathogen to cause toxic megacolon. We demonstrated that sporulation and toxin production are delayed when *C. difficile’*s proline reductase pathway is interrupted through the deletion of the *prdB* gene. Survival was enhanced in mice co-colonized with *C. sardiniense* and the *C. difficile* Δ*prdB* mutant, as *C. difficile* relies on glycolytic pathways over Stickland fermentations. The present findings support the central role of proline reductase metabolism in early pathogen growth, metabolism, and toxin, and as a potential therapeutic target against *C. difficile infection*.

## INTRODUCTION

*Clostridioides difficile* is a toxigenic, spore-forming, and anaerobic bacillus that can asymptomatically colonize the gastrointestinal tracts of humans and animals (1). Infections are commonly triggered after antibiotic depletion of metabolically competing commensals, allowing the pathogen to rapidly expand (2). Toxigenic strains produce mainly the enterotoxins TcdA and TcdB, although some produce only TcdB, and in some epidemic strains, a binary toxin, which causes severe watery diarrhea and colitis and results in additional host-derived nutrients entering the gut lumen (3).

*C. difficile* infection (CDI) is the most common healthcare-associated infection, with antibiotic exposure as the most significant risk factor (4). CDI causes substantial mortality and morbidity, with an estimated 500,000 cases per year and a total CDI-attributable expense of over $5 billion in the United States (5, 6). Recurrent CDI often follows antibiotic exposure and may require alternative treatments such as fecal microbiota transplantations and biotherapeutic agents for the resolution of infection. Therapies targeting *C. difficile’s* metabolism provide approaches to impede *C. difficile’s* colonization or expansion in the gut, to both aid in preventing primary and recurrent infections (7).

*C. difficile* primarily utilizes Stickland fermentation of leucine, glycine, and proline in reductive metabolism to support corresponding high-flux oxidative and energy-generating metabolism of substrates, including carbohydrates and other amino acids (8–10). Proline is a preferred reductive substrate that collects within the gut and originates from both the host, from collagen breakdown that releases hydroxyproline, and in the diet (11–14). Since its abundance increases after antibiotic treatment, proline was suggested to be an early nutrient source for *C. difficile* (15). The pathogen carries metabolic machinery to convert many substrates to proline (16, 17). Hydroxyproline, a major component of collagen, is converted to L-proline by 4-hydroxyproline reductase, pyrroline-5-carboxylate reductase, and racemization by proline racemase to D-proline (18). Ornithine is also readily converted to proline by ornithine cyclodeaminase (9).

The proline reductase selenoenzyme reduces D-proline to 5-aminovalerate (19), with coupling of reductive metabolism to the Rnf complex to generate proton gradients (20). Proline reductase is encoded by the *prd* operon, which consists of seven genes: *prdC*, *prdR*, *prdA*, *prdH*, *prdB*, *prdD*, *prdE,* and *prdF*. The *prdR* transcriptional regulator responds to proline to induce *prd* operon expression (16, 20). The PrdB catalytic and selenium-containing subunit is essential for enzyme activity.

Individual species of commensal *Clostridia*, including *Clostridium scindens*, *Paraclostridium bifermentans* (PBI), *Clostridium sardiniense* (CSAR), and *Peptostreptococcus anaerobius,* impact *C. difficile* colonization and subsequent infection (9, 21, 22). Similar to *C. difficile*, PBI and *P. anaerobius* demonstrate proficiency in the Stickland fermentation of proline, and these strains confer protection against *C. difficile* infection in mice through proline consumption (9, 22). Conversely, CSAR, a glycolytic, butyrate-producing species, lacks Stickland reductases but can metabolize some amino acids, including alanine and arginine, for energy. It converts arginine to ornithine via the arginine deaminase pathway, which can support *C. difficile* Stickland metabolism via separate pathways to convert ornithine to proline and to alanine. This cross-feeding mechanism contributes to the rapid lethal phenotype previously observed in mice co-colonized with CSAR and *C. difficile* through increased *C. difficile* growth, toxin production, and subsequent host tissue damage (9).

Prior studies of a *prdB* mutant in the *C. difficile* JIR8094 strain identified *in vitro* functions of the gene on proline fermentation, with no impact on upstream transformation of substrates such as hydroxyproline (19). Bouillaut et al. also demonstrated maintenance of growth *in vitro* by the *prdB* mutant, suggesting compensatory recruitment of other metabolic pathways to overcome the absence of reductive proline metabolism (16). Furthermore, *C. difficile prdB* mutants were limited in their ability to colonize clindamycin-treated fecal communities *in vitro,* thus demonstrating that proline may be critical for colonization (23). As the CD630 strain is non-infective in mice, *in vivo* colonization studies in mice constituted with donor human microbiota indicated defects in early colonization and toxin production (24, 25), though without the capacity to assess the impact on the development of symptomatic disease. However, per prior limitations to genetically manipulate mouse-infective strains, the enzyme’s role in an *in vivo* context of active infection has not been well-defined, including broader effects on pathogen metabolism and recruitment of cellular systems that modulate growth and virulence. In addition, how *C. difficile’s* proline reductase modifies metabolic interactions with host and the commensal microbiota also remains an unknown area, but one of importance given the availability of host-origin substrates such as collagen-derived hydroxyproline, and commensally produced ornithine by arginine-fermenting species, including *C. sardiniense,* and *Enterococci* (26).

We deleted the *prdB* gene in the mouse infective strain ATCC 43255 to evaluate its *in vivo* role in infection and host outcomes in highly susceptible gnotobiotic mice and in mice co-colonized with the disease-promoting commensal *C. sardiniense*. We demonstrate how the absence of proline reductase metabolism more broadly impacts the pathogen’s ability to efficiently recruit growth-promoting redox metabolism and reduce early events in pathogen colonization and growth, extending host survival but not preventing the development of severe disease. Our findings identify proline reductase’s contribution to central metabolic strategies used by *C. difficile* in early gut colonization, growth, and eventual virulence, and identify metabolic and small-molecule strategies to aid in preventive and therapeutic strategies.

## RESULTS

### Absence of reductive proline metabolism delays *C. difficile* colonization and growth in an enriched gut nutrient environment

**Fig 1:**
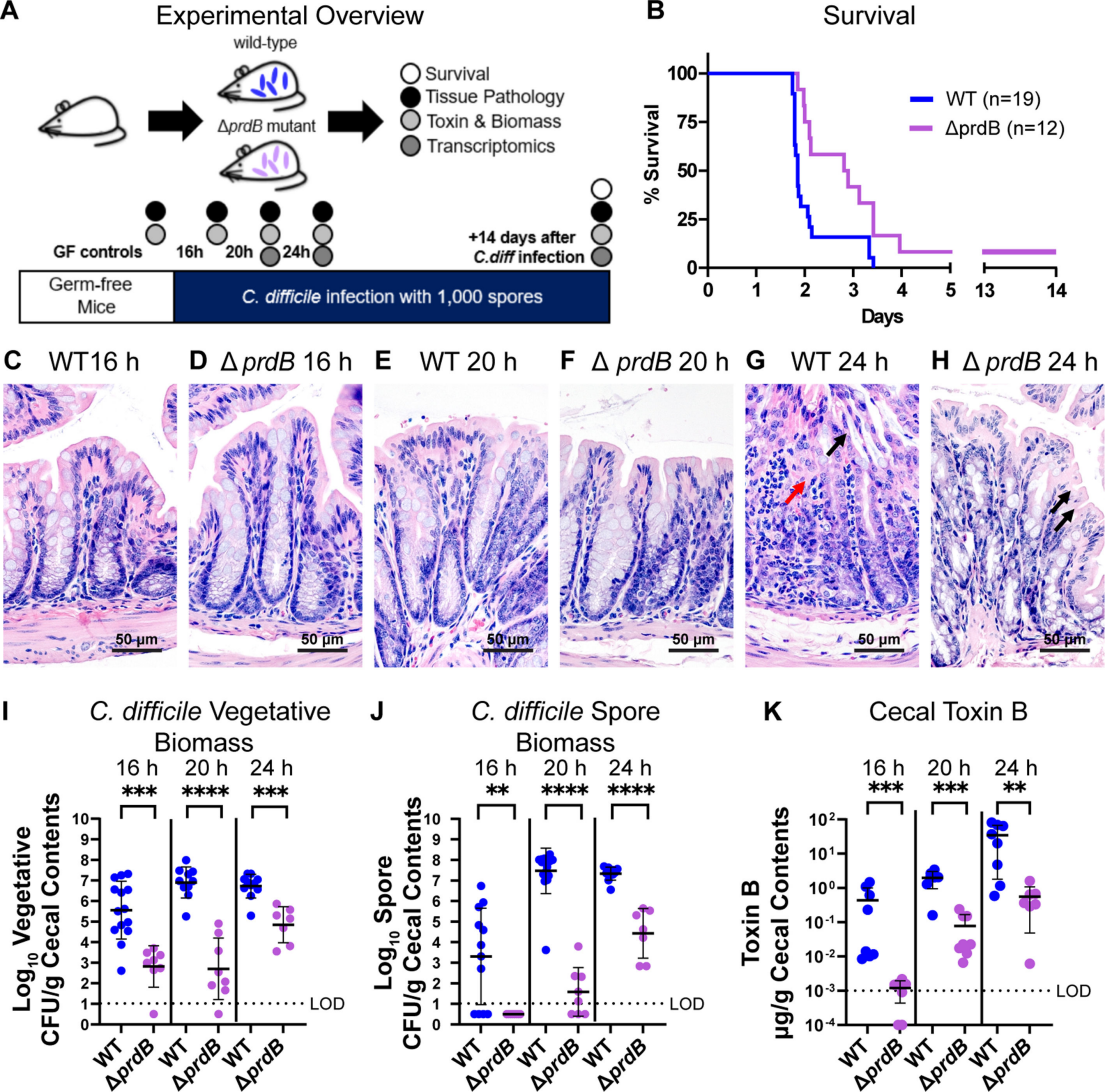
Mouse infection with a *C*. *difficile* Δ*prdB* mutant shows delayed growth and toxin production *in vivo*. (**A**) Experimental overview showing the gnotobiotic *C. difficile* infection model. (**B**) Kaplan-Meier survival curves showing delayed lethal infection in mice infected with the Δ*prdB* mutant; Mantel-Cox test, *P* = 0.007. (**C–H**) Hematoxylin and eosin (H&E)-stained colon sections. Magnification 200×, scale bar 50 μm. (**C**) Colonic mucosa from a germ-free mouse infected with WT *C. difficile* at 16 h of infection showing no signs of disease, (**D**) Healthy colonic mucosa from a germ-free mouse infected with the Δ*prdB* mutant at 16 h of infection, (**E**) Colonic mucosa from a germ-free mouse infected with WT *C. difficile* at 20 h of infection. (**F**) Colonic mucosa from a germ-free mouse infected with the Δ*prdB* mutant at 20 h of infection. (**G**) Colonic mucosa from a germ-free mouse infected with WT *C. difficile* at 24 h showing surface epithelial stranding and sloughing (black arrow) and transmural neutrophilic infiltrates (red arrow), and (**H**) colonic mucosa from a germ-free mouse infected with the Δ*prdB* mutant at 24 h of infection showing slight disease and vacuolation of surface colonocytes (black arrows). (**I**) Log_10_ *C. difficile* vegetative biomass. (**J**) Log_10_ *C. difficile* spore biomass. (**K**) Cecal toxin B (μg toxin/g cecal contents). Bars show mean and standard deviation. Limit of detection is denoted by LOD. Mann-Whitney significance values: ∗∗ 0.001 < *P* ≤ .01, ∗∗∗ 0.0001 < *P* ≤ .001.

Swiss-Webster gnotobiotic mice infected with 1,000 spores of the wild-type (WT) *C. difficile* ATCC 43255 strain succumbed to disease within 72 h post-infection (Fig. 1A and B). In contrast, mice infected with the isogenic Δ*prdB* mutant strain (Δ*prdB*) showed extended host survival (Mantel-Cox *P* < 0.01) that was evident by 24 h, with one mouse surviving to 14 days post-infection. Median survival times for the WT and Δ*prdB* groups were 44 and 68 h, respectively (Fig. 1B).

The colonic mucosa in the WT and ∆*prdB*-infected mice showed normal mucosa at 16 h of infection (compare Fig. 1C and D). However, over 20–24 h of infection, vacuolation and stranding of surface colonocytes also occurred within the infected mice (Fig. 1E through H), while more severe surface epithelial sloughing and transmural neutrophilic infiltrates were visible by 24 h post-infection with the WT strain alone (Fig. 1G). At both 20 and 24 h of infection, WT showed more damage to the colonic mucosa.

Assessments of cecal toxin B and *C. difficile* vegetative and spore biomass demonstrated a colonization defect in the Δ*prdB* mutant with decreased vegetative and spore biomass, and delayed production of toxin B in cecal contents throughout infection (Fig. 1I through K). In agreement, we observed *in vitro* a significant reduction of the vegetative cell growth in rich TY medium in the Δ*prdB* mutant compared to the WT (Fig. S1a). This is consistent with recent data showing that a *prdA* mutant of the *C. difficile* strain 630Δerm, affected in proline reductase activity, exhibits a reduction in vegetative and spore biomass (27). In addition, although not significant, a trend toward an early (8 h) delay in toxin A secretion in the Δ*prdB* mutant, although the amount of toxin produced appears to be identical to that of the WT strain. However, the quantity of toxin A produced and secreted later (24 h) in the Δ*prdB* mutant, similar to the WT strain, is consistent with the lethal capacity of the Δ*prdB* mutant (Fig. S1b).

### Removal of reductive proline metabolism broadly disrupts *C. difficile’s* metabolic strategies to colonize and adapt to changing gut nutrient conditions

**Fig 2:**
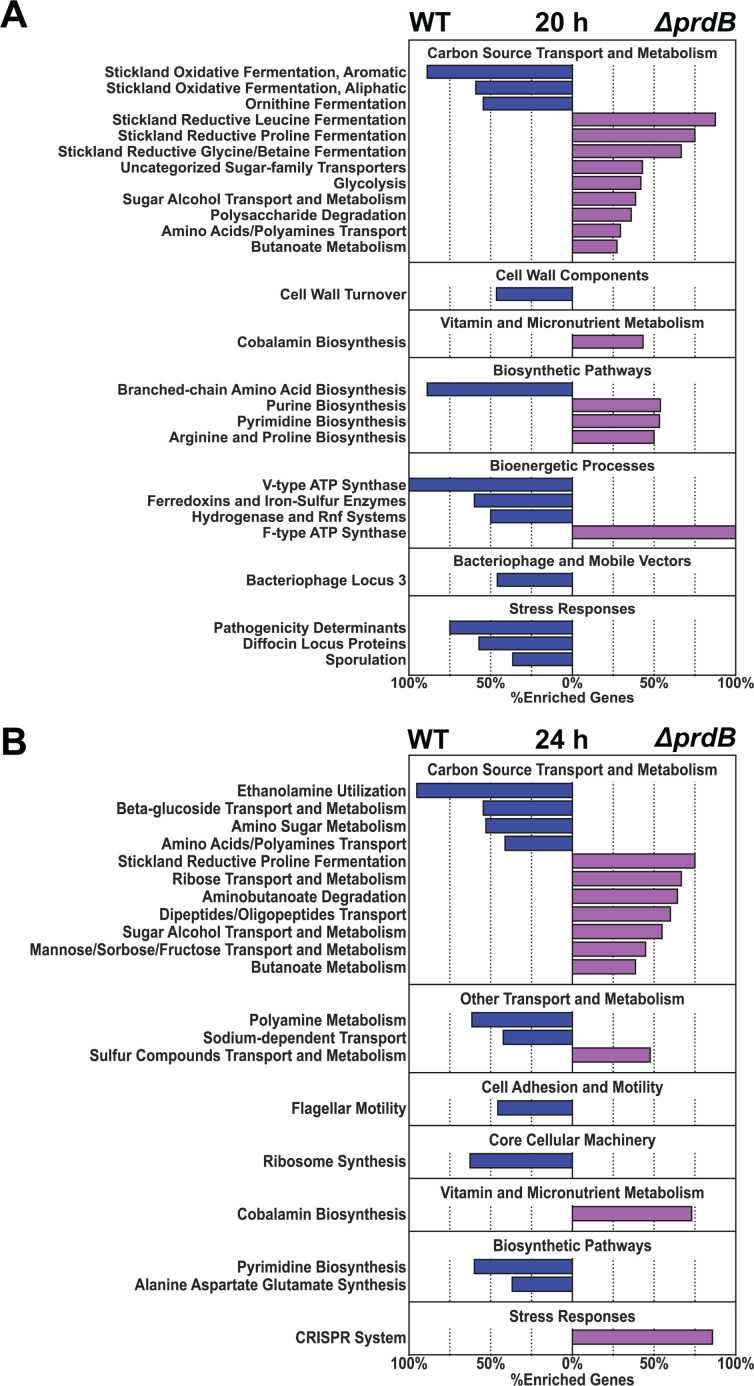
*C*. *difficile* Δ*prdB* mutant induces metabolic disruptions *in vivo,* reflected by the pathogen’s global gene expression**.** Metatranscriptomic analyses of *C. difficile’s* metabolic, core cellular machinery, and cellular stress systems that were significantly enriched in mice challenged with wild-type *C. difficile* (WT, blue) or Δ*prdB* mutant *C. difficile* (Δ*prdB,* purple). Horizontal bars indicate the percentage of enriched genes with a Benjamini-Hochberg-corrected *P*-value ≤ 0.05. Headings above the bars show general pathway categories with subpathways on the left. (**A**) Enriched pathways at 20 h post-infection with WT or Δ*prdB C. difficile.* (**B**) Enriched pathways at 24 h post-infection with WT or Δ*prdB C. difficile*.

**Fig 3:**
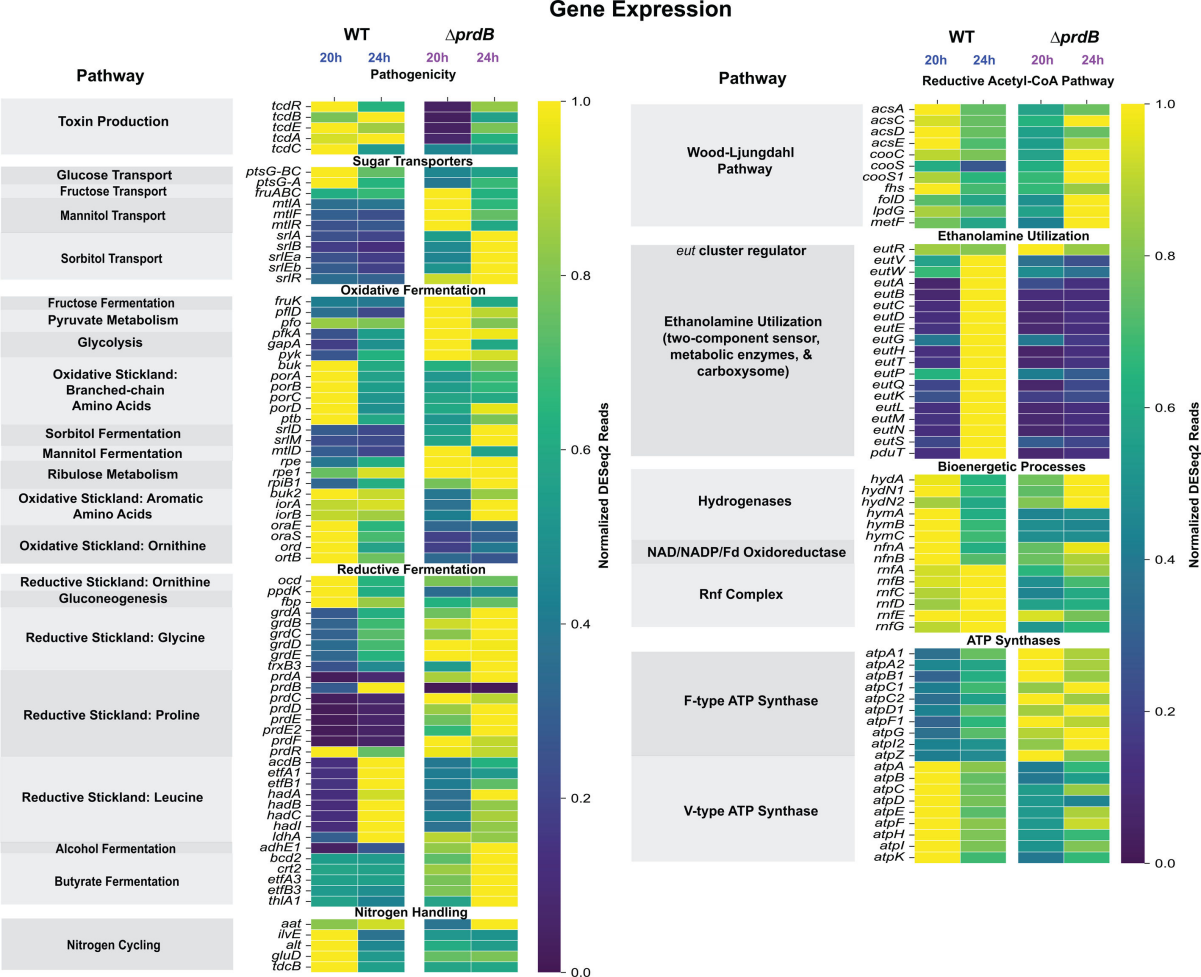
*C*. *difficile* Δ*prdB* mutant fails to induce energy-generating oxidative Stickland metabolism and adjusts to alternative reductive amino acid and oxidative carbohydrate fermentation systems**.** Heatmap colors indicate the proportion of maximum gene expression for each gene as normalized DESeq2 reads (scale 0–1). Timepoints are indicated at 20 and 24 h post-infection with either the *C. difficile* wild-type (WT, blue) ATCC 43255 strain or Δ*prdB* mutant (Δ*prdB,* purple). Pathogenicity and metabolic categories (e.g., oxidative fermentation) are listed between each break in the heatmap, and pathways are labeled in the gray boxes to the left of the gene names.

*In vivo* analyses of *C. difficile* gene expression in cecal contents identified multiple metabolic disruptions over the course of infection with broader impacts on additional cellular systems (Fig. 2). In contrast to the wild-type strain, over 20–24 h of colonization, the Δ*prdB* mutant failed to induce its Stickland oxidative fermentation and ornithine fermentations pathways that generate alanine and proline (Fig. 2A and Fig.3). To compensate, the Δ*prdB* mutant induced the expression of reductive Stickland leucine and glycine metabolism (Fig. 2A), in addition to the other *prd* operon genes, though with undetectable *prdB* (Fig. 3).

The Δ*prdB* mutant compensated for the defects in its Stickland redox metabolism by recruiting pathways for sugar alcohol metabolism, inducing mannitol transport and fermentation at 20 h, followed by sorbitol metabolism at 24 h post-infection, respectively (Fig. 3). Fructose transport and fermentation genes, *fruABC* and *fruK,* were also upregulated at 20 h post-infection with the ∆*prdB* mutant (Fig. 3).

Ethanolamine, a fermentable substrate released from damaged mucosa and from host membrane-associated phosphatidyl-ethanolamine lipids (28), was induced by 24 h of infection with the WT (Fig. 2B). In contrast, the Δ*prdB* mutant consistently relied on glycolytic and mixed acid fermentation reactions that produce acetate and butyrate, particularly over 20–24 h of infection, and showed limited metabolic adaptations inducing only pathways for the transport and metabolism of ribose, transformation of mannose for glycolysis, oxidative Stickland pathways for aromatic amino acids, and upregulation of pathways for cobalamin biosynthesis (Fig. 2B) (29). *In vitro*, we observed that the Δ*prdB* mutant grows similarly to the WT in a defined *C. difficile* minimal medium (CDMM) with no glucose but better than the WT in the presence of glucose, probably by favoring glycolysis flux in the medium (Fig. S2). The Δ*prdB* mutant grew even better than the WT when leucine was added to the medium, suggesting that the Δ*prdB* mutant can compensate for the absence of proline metabolism by using other metabolic pathways such as the leucine metabolic pathway encoded by the *hadAIBC-acdB-etfBA* operon (Fig. S2).

With the underlying defects in Stickland metabolism, the Δ*prdB* mutant showed reduced expression of its alanine transaminase (UAB_RS0207590) that integrates glycolytic and de-aminating Stickland metabolism to aid in the generation of anabolic substrates for protein and cell wall synthesis (Fig. 3) (30). Expression of genes associated with bioenergetic processes, including hydrogenases, V-type ATP synthases, and Rnf complex, was also affected in the mutant (Fig. 3).

The combined *in vivo* metatranscriptomics, with host and *C. difficile* phenotypes, illustrated how sole disruption of the *prdB* gene and reductive proline metabolism broadly impacted the pathogen’s early recruitment of efficient redox metabolism and subsequent phenotypes on delayed growth, sporulation, and toxin production. Despite these delays in growth and virulence, the pathogen produced sufficient toxin to cause severe disease in monocolonized mice.

### Absence of *C. difficile’s* reductive proline metabolism enhances survival of mice co-colonized with the disease-promoting commensal, *C*. *sardiniense*

**Fig 4:**
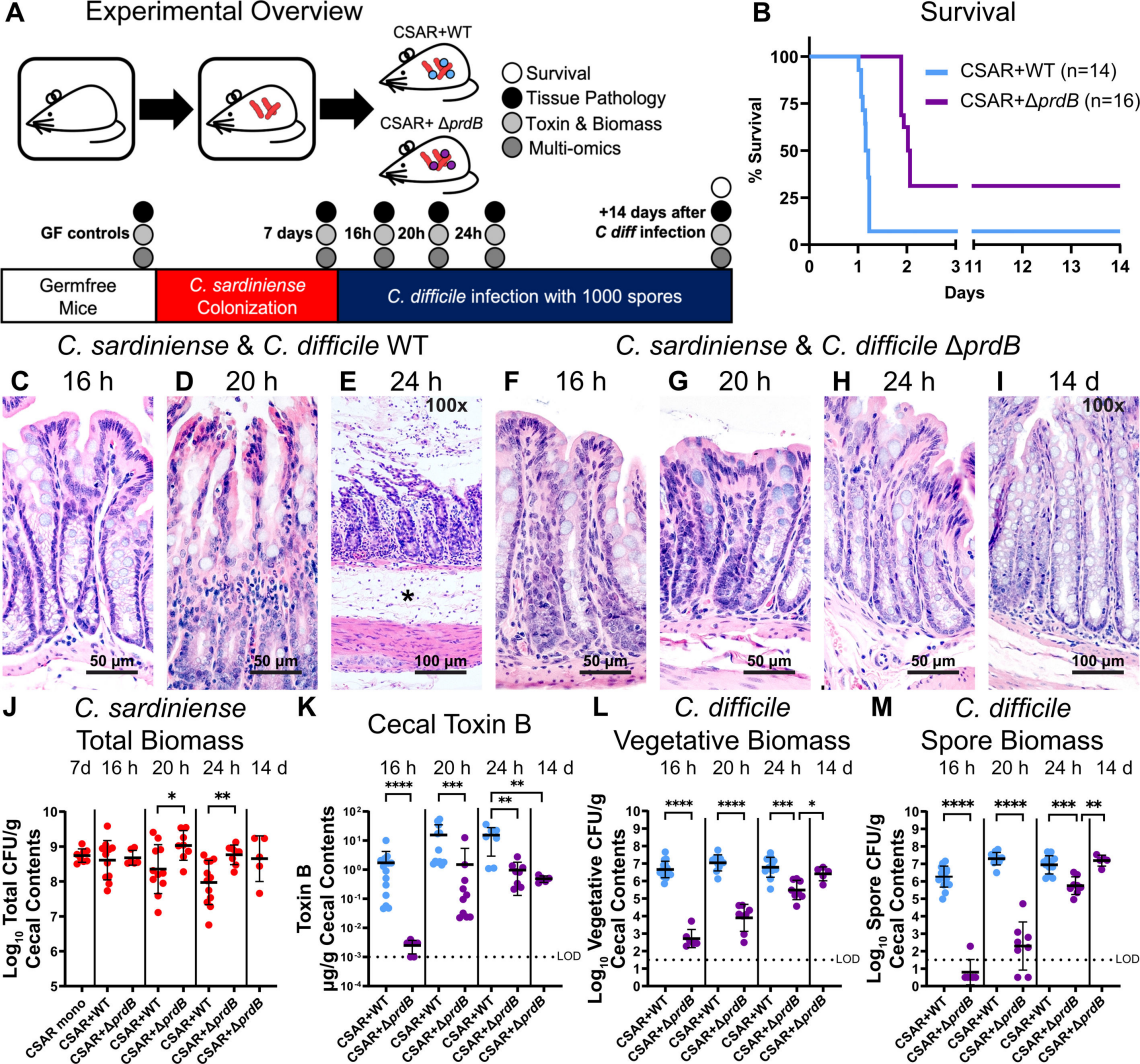
Mouse inoculation with *C*. *sardiniense* and subsequent infection with *C*. *difficile* Δ*prdB* mutant shows delayed growth and toxin production *in vivo*. (**A**) Experimental overview showing *C. sardiniense* establishment and subsequent *C. difficile* infection model with and without the Δ*prdB* mutant. (**B**) Kaplan-Meier survival curves showing delayed infection in mice infected with the *C. sardiniense*-Δ*prdB* mutant; Mantel-Cox test, *P* < 0.001. (**C–K**) H&E-stained colon sections. Magnification 200×, scale bar 50 μm, except subfigures **E** and** I**, which were imaged at 100× magnification, scale bar 100 µm. (**C**) Colonic mucosa from a germ-free mouse colonized with *C. sardiniense* and infected with wild-type *C. difficile* at 16 h of infection showing no signs of disease, (**D**) colonic mucosa from a germ-free mouse colonized with *C. sardiniense* and infected with wild-type *C. difficile* at 20 h with neutrophil infiltrates (black arrow), (**E**) colonic mucosa from a germ-free mouse colonized with *C. sardiniense* and infected with wild-type *C. difficile* at 24 h with neutrophil infiltrates, severe submucosal edema, and epithelial denudation (asterisk)**,** (**F**) colonic mucosa from a germ-free mouse colonized with *C. sardiniense* and infected with *C. difficile* Δ*prdB* mutant at 16 h showing no signs of disease, (**G**) colonic mucosa from a germ-free mouse colonized with *C. sardiniense* and infected with *C. difficile* Δ*prdB* mutant at 20 h, (**H**) colonic mucosa from a germ-free mouse colonized with *C. sardiniense* and infected with *C. difficile* Δ*prdB* mutant at 24 h with slight epithelial stranding, (**I**) colonic mucosa from a germ-free mouse colonized with *C. sardiniense* and infected with *C. difficile* Δ*prdB* mutant at 14 days post-infection. (**J**) Log_10_ *C. sardiniense* total biomass over time. (**K**) Cecal toxin B (µg toxin/g cecal contents). (**L**) Log_10_ *C. difficile* vegetative biomass. (**M**) Log_10_ *C. difficile* spore biomass. Bars show mean and standard deviation. Limit of detection is denoted by LOD. Mann-Whitney significance values: ∗∗ 0.001 < *P* ≤ .01, ∗∗∗ 0.0001 < *P* ≤ .001.

Swiss-Webster gnotobiotic mice colonized with *C. sardiniense* DSM 599 (CSAR) for 7 days and infected with 1,000 spores of the *C. difficile* ATCC 43255 strain (WT) rapidly succumbed to lethal infection within 72 h (Fig. 4A and B), with only one of fourteen (7.1%) mice surviving to 14 days post-infection, whereas mice infected with the Δ*prdB* mutant showed extended host survival, with 31% of mice surviving to 14 d post-infection (Fig. 4B; *P* < 0.0001).

The colonic mucosa in the CSAR + WT-infected mice showed transmural neutrophil recruitment into the mucosa with severe submucosal edema by 20 h post-infection with epithelial denudation by 24 h post-infection (compare Fig. 4C through E). In contrast, mice challenged with the Δ*prdB* mutant demonstrated milder colonic damage by 20 h post-infection with less severe neutrophil infiltration and lack of submucosal edema by 24 h post-infection (compare Fig. 4C through H).

**Fig 5:**
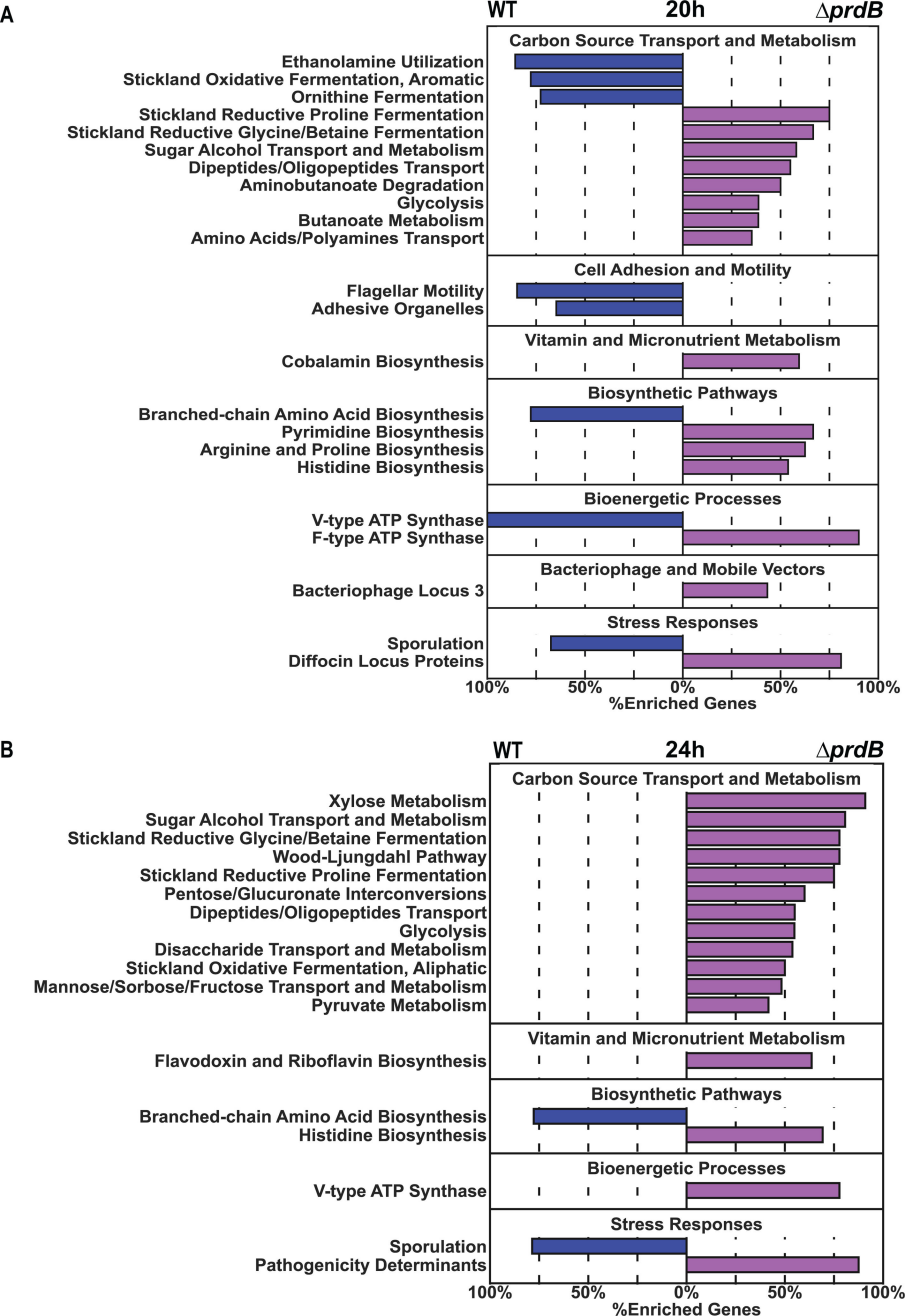
*C*. *difficile* Δ*prdB* mutant induces metabolic disruptions *in vivo,* reflected by the pathogen’s global gene expression in mice co-colonized with *C*. *sardiniense***.** Metatranscriptomic analyses of *C. difficile’s* metabolic, core cellular machinery, and cellular stress systems that were significantly enriched in mice co-colonized with *C. sardiniense* and wild-type *C. difficile* (WT, blue) or Δ*prdB* mutant *C. difficile* (Δ*prdB,* purple). Horizontal bars indicate the percentage of enriched genes with a Benjamini-Hochberg-corrected *P*-value ≤ 0.05. Headings above the bars show general pathway categories with subpathways on the left. (**A**) Enriched pathways at 20 h post-infection with *C. sardiniense* and *C. difficil*e WT or Δ*prdB.* (**B**) Enriched pathways at 24 h post-infection with *C. sardiniense* and *C. difficile* WT or Δ*prdB .*

**Fig 6:**
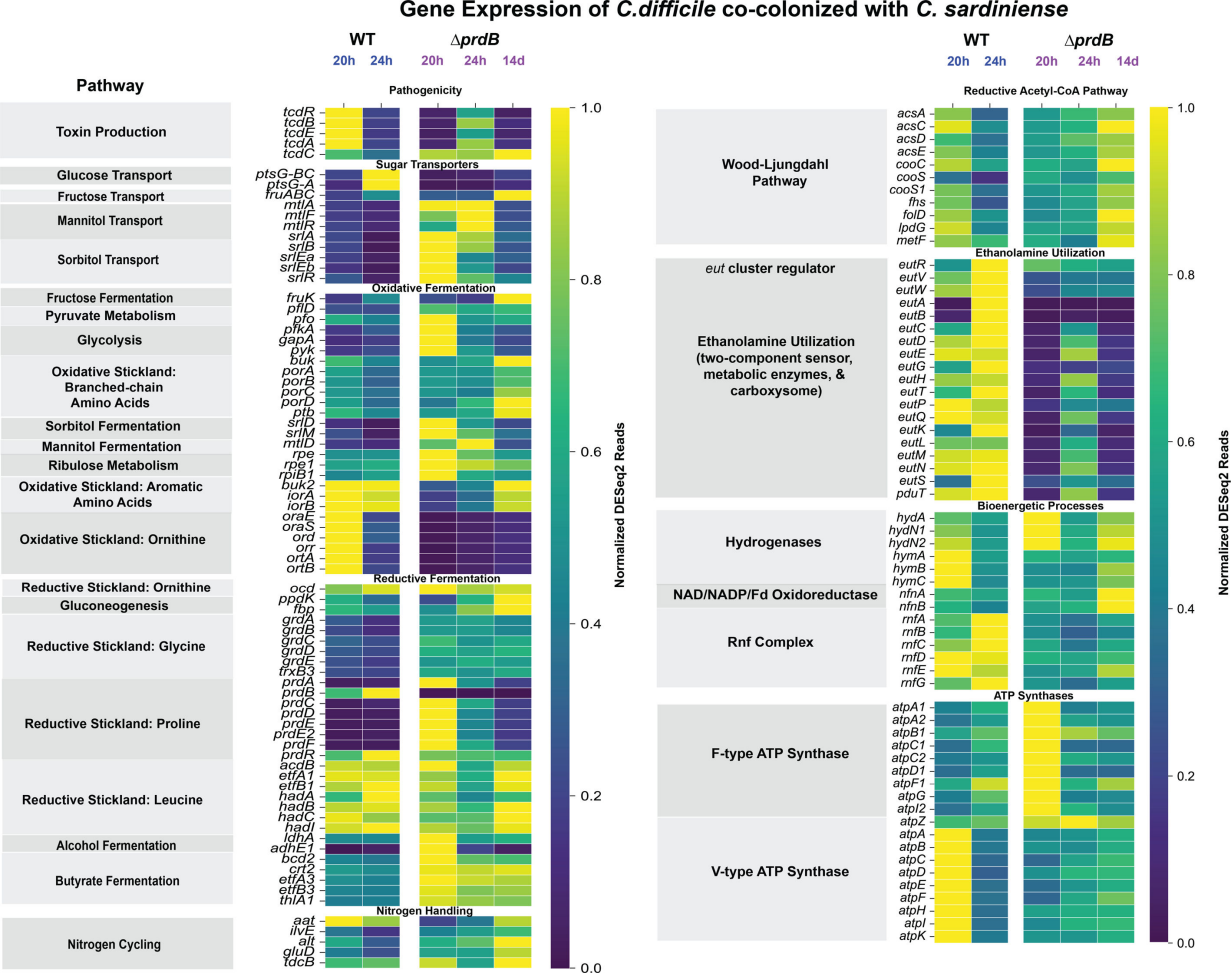
In co-colonization with *C*. *sardiniense,* the *C*. *difficile* Δ*prdB* mutant represses PaLoc operon genes and induces glycolytic pathways over alternative oxidative Stickland fermentation. Heatmap colors indicate the proportion of maximum gene expression for each gene as normalized DESeq2 reads (scale 0–1). Timepoints are indicated at 20 and 24 h post-infection with either the *C. difficile* wild-type (WT; blue) ATCC 43255 strain or Δ*prdB* mutant (Δ*prdB*; purple) co-colonized with *C. sardiniense*. Pathogenicity and metabolic categories are listed between each break in the heatmap, and pathways are labeled in the gray boxes to the left of the gene names.

In comparison to CSAR + WT mice, cecal toxin, vegetative, and spore biomasses were lower in CSAR + Δ*prdB* mice, demonstrating delayed colonization with subsequent delay of infection (Fig. 4K through M). CSAR growth is similar in mice co-colonized with WT or Δ*prdB*, and increases to some extent at 20–24 h post-infection in mice co-colonized with CSAR + Δ*prdB* (Fig. 4J). Metatranscriptomic analyses further demonstrated that *in vivo* sporulation genes were downregulated throughout infection and pathogen determinant genes upregulated in mice co-colonized with CSAR + Δ*prdB* by 24 h post-infection (Fig. 5). Cecal toxin concentrations increased over time in CSAR + Δ*prd*B mice and were maintained by 14 days post-infection but did not achieve the acute concentrations observed in CSAR + WT mice (Fig. 4K). In mice co-colonized with CSAR, the *C. difficile* WT strain demonstrated an induction of genes within the Pathogenicity Locus (PaLoc) operon at 20 h post-infection, while *C. difficile* Δ*prdB* induced PaLoc genes at 24 h (Fig. 6).

In contrast to the WT strain, the presence of CSAR in mice induces alternative Stickland fermentation pathways in the Δ*prdB* mutant as well as the transport and metabolism of sugar alcohols, and, in part, glycolysis (Fig. 5 and 6). While the Δ*prdB* mutant induced the reductive leucine pathway over the glycine reductive pathway throughout the infection, carbohydrate metabolism genes remained upregulated. The Δ*prdB* mutant downregulated ornithine and aromatic Stickland oxidative fermentation genes in comparison to *C. difficile* WT strain in the presence of CSAR. Genes in the reductive leucine pathway, fructose transport, and gluconeogenesis were enriched in five of the sixteen Δ*prdB* mutant mice that survived to 14 days post-infection and co-colonized with CSAR (Fig. 6).

**Fig 7:**
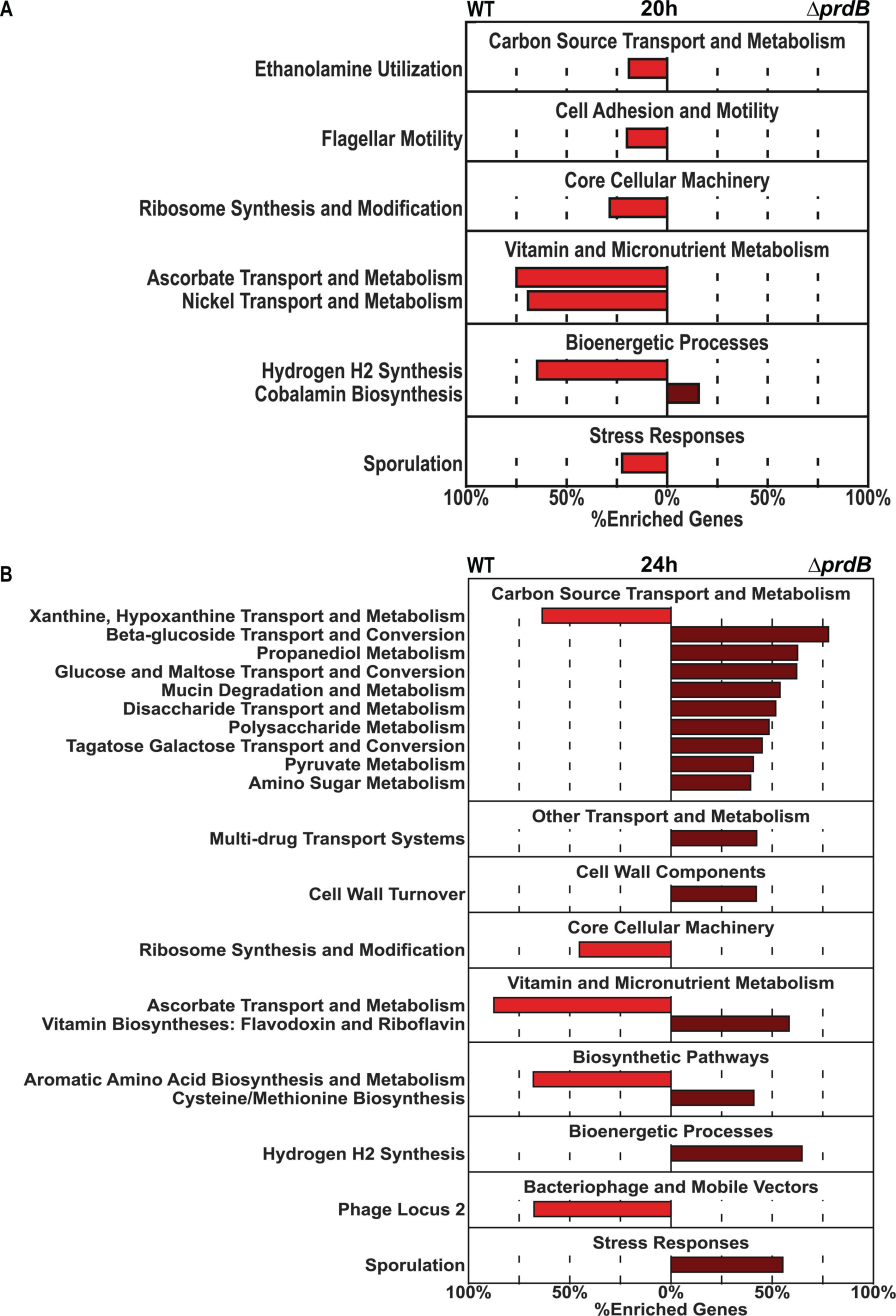
*C***.** *sardiniense* modulates its metabolism to compete with *C*. *difficile in vivo*. Metatranscriptomic analyses of *C. sardiniense’s* metabolic, core cellular machinery, and cellular stress systems that were significantly enriched in mice co-colonized with *C. sardiniense* and *C. difficile* wild type (CSAR + WT, red) or *C. difficile* Δ*prdB* mutant (CSAR + Δ*prdB,* dark red). Horizontal bars indicate the percentage of enriched genes with a Benjamini-Hochberg-corrected *P*-value ≤ 0.05. Headings above the bars show general pathway categories with subpathways on the left. (**A**) Enriched pathways at 20 h post-infection with CSAR + WT or CSAR + Δ*prdB.* (**B**) Enriched pathways at 24 h post-infection with CSAR + WT or CSAR + Δ*prdB*.

When co-colonized with the Δ*prdB* mutant*,* CSAR downregulated its own genes of sporulation and ethanolamine utilization and upregulated genes of cobalamin biosynthesis at 20 h post-infection (Fig. 7). CSAR upregulated genes of disaccharide degradation and metabolism with enrichment of the cellobiose-specific phosphotransferase system (PTS), mucin degradation with an enrichment of glycosyl hydrolases, sporulation, glucose, and mannitol transport with the enrichment of the maltodextrin ABC transporter, and amino sugar metabolism in mice co-colonized with the Δ*prdB* mutant during late infection. Xanthine, ascorbate, and aromatic amino acid metabolism genes were downregulated by CSAR when infected with the Δ*prdB* mutant.

## DISCUSSION

We generated a Δ*prdB C. difficile* mutant in the mouse-infective strain ATCC 43255 to define the *in vivo* functions of reductive proline metabolism in the pathogen’s broader redox metabolism, growth, and virulence. In both experiments, with and without CSAR co-colonization, the Δ*prdB* mutant demonstrated delayed colonization, growth, and toxin production, which extended host survival. While gnotobiotic mice monocolonized with the Δ*prdB* mutant strain ultimately succumbed to infection, our findings demonstrated how the absence of reductive proline metabolism impacted *C. difficile’s* capacity to efficiently recruit and harness its redox metabolism for energy and anabolic substrates during early stages of gut colonization. Further extension of survival observed in 31% of mice co-colonized with the Δ*prdB* mutant and CSAR was observed as toxin B concentrations remained stable from 24 h to 14 days post-infection and never reached those concentrations occurring in CSAR + WT co-colonized mice (Fig. 4K). In both colonization states, the Δ*prdB* mutant downregulated the expression of its PaLoc genes *tcdA*, *tcdB*, *tcdE,* and *tcdR* in comparison to the wild-type strain, leading to the delay in symptoms and enhanced survival. However, even by 24 h, as host symptoms progressed, the Δ*prdB* mutant still failed to adequately use some available gut nutrients in monocolonized gnotobiotic mice, like ethanolamine, from damaged host mucosa by 24 h post-infection (Fig. 3) (28).

With the disruption of proline reductase metabolism, the pathogen recruited alternative Stickland reductive pathways for leucine and glycine fermentation in Δ*prdB* monocolonized mice (16). Notably, the mutant alone failed to induce ornithine metabolism genes during early stages of colonization, including those converting ornithine to alanine, a key growth-promoting substrate, as well as to proline by ornithine cyclodeaminase (31). While the ∆*prdB* mutant recruited pathways for glycolysis *in vivo*, pyruvate metabolism, and subsequent butyrate metabolism, it did not concomitantly induce its alanine transaminase to support efficient amino group nitrogen handling from deaminating Stickland metabolism, including for reductive glycine and leucine metabolism.

The absence of reductive proline metabolism effectively converted *C. difficile* into a metabolically more permissive pathogen in monocolonized mice, but one lacking optimized redox metabolism to subsist more efficiently on carbohydrate substrates. Lacking efficient oxidative and reductive Stickland metabolism during early stages of monocolonization, the Δ*prdB* mutant turned to alternative carbohydrate sources, including sugar alcohols, fructose, mannose, and ribose. This is consistent with the *in vitro* rescue of growth of the mutant by glucose addition to CDMM, further demonstrating the adaptability of the mutant to switch from Stickland to carbohydrate metabolism. While mannitol is from dietary sources (9, 32, 33), sorbitol can be derived from dietary and host sources, including from immune cells via the activity of aldose reductase, an enzyme that is upregulated during host inflammation (34). In monocolonized mice, the mutant also induced multiple cobalamin biosynthetic operon genes to support cobalamin-dependent pathways for these recruited metabolic pathways, in addition to pathways for methionine and DNA synthesis (35).

The ∆*prdB* mutant in the absence of proline metabolism recruited pathways to support glycolytic pathways in contrast to ornithine metabolism, likely in part due to the inability of the pathogen to utilize the proline generated by the conversion of ornithine by ornithine cyclodeaminase. While the mutant could convert ornithine to alanine for Stickland fermentation, it alternatively expressed glycolytic pathways, including sugar alcohol metabolism. CSAR remained metabolically competitive by recruiting alternative carbon metabolism pathways to the mutant, including polysaccharide degradation pathways with maltodextrin transport and metabolism. The ability of CSAR to metabolize maltodextrin may be beneficial to host inflammation, as this starch derivative exacerbates intestinal inflammation through stress on the endoplasmic reticulum of intestinal epithelial cells with subsequent reduction of mucus. Furthermore, CSAR’s competitive use of cellobiose over the ∆*prdB* mutant may have contributed to the observed delay in *C. difficile* sporulation, as cellobiose uptake inhibition was previously shown to decrease *C. difficile* sporulation efficiency (36).

CSAR’s induction of mucin degradation generates fermentable carbohydrates that could be used by the ∆*prdB* mutant but also for its own use, thus demonstrating the ability of this commensal to compete metabolically with the pathogen. Previously, *C. difficile* growth and expansion were aided by a microbiota-induced increase in sialic acid concentrations (37). Thus, the upregulation of mucin degradation, including sialidases by CSAR, which leads to the cleavage of sialic acid residues from the glycosylated protein, may provide sialic acid to support ∆*prdB* mutant growth. However, the ∆*prdB* mutant demonstrated delayed germination and growth with CSAR in comparison to the WT strain. Commensal *Clostridium* spp. were previously not predicted to degrade mucins like the pathogen *Clostridium perfringens* but were suspected to utilize the mucins to enhance commensal growth (38). By 24 h of infection with the ∆*prdB* mutant, CSAR upregulated sporulation that coincided with an increase in total CSAR biomass that persisted to 14 days post-infection, while the ∆*prdB* mutant downregulated sporulation. Maintenance of the CSAR biomass during infection further enabled CSAR to metabolically compete with the ∆*prdB* mutant.

Our findings define complex mechanisms by which reductive proline metabolism enables *C. difficile’s* initial colonization, growth, and toxin production by supporting efficient induction of broader energy-generating and anabolic pathways. While the Δ*prdB* mutant’s metabolic shifts to glycolytic and mixed acid fermentations supported eventual growth and pathogenesis in a highly enriched gut nutrient environment, it remained less able to effectively harness preferred amino acids and newly available nutrients, such as ethanolamine, for energy and growth as infection progressed. Given the presence of proline reductase metabolism in other *Clostridial* pathogens, such as *Clostridium botulinum,* strategies to disrupt this pathway provide opportunities to leverage metabolic approaches in treatment and for disease prevention.

## MATERIALS AND METHODS

### *In vitro* growth conditions and general methods

*C. difficile* strains were grown in an anaerobic chamber (Jacomex, Dagneux, France) under an anaerobic atmosphere (5% H_2_, 5% CO_2_, and 90% N_2_) in TY (39) or a glucose-free medium derived from CDMM with the following composition: Oxoid casein hydrolysate (10 mg/mL) (Thermo Fisher Scientific, Waltham, MA), L-tryptophan (0.5 mg/mL), L-cysteine (0.01 mg/mL), L-leucine (0.0033 mg/mL), L-isoleucine (0.0033 mg/mL), L-valine (0.0033 mg/mL), Na_2_HPO_4_ (5 mg/mL), NaHCO_3_ (5 mg/mL), KH_2_PO_4_ (0.9 mg/mL), NaCl (0.9 mg/mL), (NH_4_)_2_SO_4_ (0.04 mg/mL), CaCl_2_·2H_2_O (0.026 mg/mL), MgCl_2_·6H_2_O (0.02 mg/mL), MnCl_2_·4H_2_O (0.01 mg/mL), CoCl_2_·6 H_2_O (0.001 mg/mL), FeSO_4_·7H_2_O (0.004 mg/mL), D-biotin (0.001 mg/mL), calcium-D-pantothenate (0.001 mg/mL), and pyridoxine (0.1 µg/mL). This medium was supplemented with glucose or leucine when necessary (8). Growth curves were obtained in 96-well microplates at 37°C in TY, CDMM with no glucose, or supplemented with 0.5% glucose or with 0.5% glucose and 30 mM leucine, and automatic recording of OD_600_ (optical density at 600 nm) was done every 30 min using a CLARIOstar plate reader (BMG Labtech, Ortenberg, Germany).

### Construction of the *ΔprdB* mutant in ATCC 43255

The ∆*prdB* mutant in the *C. difficile* ATCC 43255 strain background was obtained by allele-coupled exchange using the pseudo-suicide plasmid pMSR0 as described by Peltier et al. (2020) (40). Briefly, homology arms of both upstream and downstream locations of the *prdB* gene were PCR amplified with primers of upstream (F- TTTTTTGTTACCCTAAGTTTGGTACATTAGGAGCTCAAC, R-AAACGTGAGCCCTATCCATTATAATTCTACCTC) and downstream (F- AATGGATAGGGCTCACGTTTAAAATGTAATTTATATTTG, R- AGATTATCAAAAAGGAGTTTGGAGATATAATCATAGGTACATTTC) regions of the *prdB* genes from genomic DNA of *C. difficile* ATCC 43255. Then purified PCR products were directly cloned into the pMSR0 vector using NEBuilder HiFi DNA Assembly (New England Biolabs, Ipswich, MA). The pMSR0-derived plasmids containing the allele exchange cassettes of the target genes were transformed into *E. coli* strain NEB10β (New England Biolabs, Ipswich, MA) and verified by sequencing. Plasmids were then transformed into *E. coli* HB101 (RP4) and transferred by conjugation into strain ATCC 43255. The allele exchange protocol was similar to that used for the *codA*-mediated allele exchange (40) except that counter-selection was based on the inducible expression of the *CD2517.1* toxin gene. Briefly, 1 mL of *E. coli* HB101 (RP4) overnight culture harboring a plasmid was washed in PBS, and the cell pellet was resuspended in a 200 μL volume of overnight *C. difficile* culture and spotted onto brain heart infusion (BHI) agar (Thermo Fisher Scientific, Waltham, MA). After 24 h of incubation, the conjugation mating mixture was recovered into 600 μL of PBS, and 180 μL of the suspension was plated on BHI agar supplemented with cefoxitin (25 μg/mL), cycloserine (250 μg/mL), and thiamphenicol (7.5 μg/mL) (Millipore-Sigma, St. Louis, MO). Faster-growing single cross-over integrants were selected based on the size of the colonies. A large colony was re-streaked twice on BHI with thiamphenicol to ensure the purity of the single crossover integrant. Colonies that underwent the second recombination event were then selected on BHI plates containing 100 ng/mL anhydrotetracycline (ATc) (Millipore-Sigma, St. Louis, MO). In the presence of ATc, cells in which the plasmid is still present produce CD2517.1 at toxic levels and do not form colonies. Growing colonies were then tested by PCR with upstream and downstream primers of the *prdB* gene (F-ATCC43255 GAAATAATGGGACAAGGTG and R- ATCC43255 CCTTTGTTCTTAAAACCCC) for the presence of the expected deletion.

### *In vivo* infection studies in gnotobiotic mice

*C. difficile* bacterial spores for animal experiments were prepared by growing a lawn from a bacterial isolate on several Brucella agar plates (Remel, Lenexa, KS), then allowing these plates to incubate anaerobically at 37 °C for 14 days to allow drying and promote spore formation. The bacterial lawns were harvested by cotton swab in sterile PBS (Thermo Fisher Scientific, Waltham, MA), then mixed 1:1 with 95% ethanol to kill non-spores. The spores were then washed two times and resuspended in PBS for storage using centrifugation at 5,000 × *g* for 10 min to pellet the spores. Spore preparation titers were assessed by plating serial dilutions onto *C. difficile*-selective CHROMID agar (Biomérieux, Durham, NC) for *C. difficile.*

Equal numbers of male and female Swiss Webster gnotobiotic mice at 6–7 weeks old were singly housed in sterile OptiMice cages (Animal Care Systems, Centennial, CO) (41). Germ-free mice were challenged with either 1 × 10^3^ spores of wild-type ATCC 43255 strain or the isogenic Δ*prdB* mutant for the mono-associated studies (Fig. 1A). For co-colonization studies with *C. sardiniense* DSM 599 (CSAR), mice were administered 4 × 10^6^ CFU of *C. sardiniense* from a flash frozen BHI culture by oral gavage to allow for colonization over 7 days prior to challenge with *C. difficile*. After 7 days, fecal samples were collected to confirm colonization with only *C. sardiniense* or maintenance of germ-free status (Fig. 4A). In both experiments, mouse survival up to 14 days post-infection with *C. difficile* was monitored for disease progression. Body condition scoring was utilized to assess host health status and disease progression (42). Death was not used as an endpoint for disease.

In timepoint studies at 16, 20, and 24 h post-challenge, cecal contents were immediately collected and frozen in liquid nitrogen for downstream biomass counts, transcriptomics, and toxin B ELISA analyses, while the gastrointestinal tract was fixed in formalin for paraffin embedding and H&E staining of 5 mm sections.

### Biomass quantification of *C. difficile* and *C. sardiniense*

Previously, flash-frozen cecal contents were weighed in 1.5 mL tubes and kept frozen on a cold block prior to transfer into a Coy anaerobic chamber (Coy Labs, Grass Lake, MI) at 37°C with an atmosphere of 2.5% H_2_, 10% CO_2_, and 87.5% N_2_. Samples were homogenized in 1 mL of pre-reduced 1× phosphate-buffered saline (PBS) with 0.05% cysteine as a reducing agent (Millipore-Sigma, St. Louis, MO), serially diluted, and plated in triplicate to *C. difficile*-selective CHROMID agar (Biomérieux, Durham, NC) or Brucella agar for *C. sardiniense* (Remel, Lenexa, KS). *C. difficile* colonies were counted at 72 h post-incubation and identified as large black colonies. *C. sardiniense* colonies were counted at 24 h post-incubation and identified as small, round, beta-hemolytic colonies (9).

*C. difficile* spores were isolated from pre-weighed, diluted, and homogenized cecal contents by exposure to 50% ethanol for 1 h and subsequently centrifuged to form a pellet. The pellet was washed twice with 1× PBS to remove the ethanol prior to serial diluting and plating the samples in triplicate on CHROMID agar. *C. difficile* colonies from the spore fraction were identified and counted as described above.

### Histopathology

Formalin-fixed colon tissues from germ-free, *C. difficile* mono-colonized, and *C. difficile-C. sardiniense* co-colonized animals were blocked, paraffin-embedded, and cut into 5 mm sections for staining with H&E stain as previously described (43). Slides were visualized using a Nikon Eclipse E600 microscope (Nikon, Melville, NY) to assess features of epithelial damage, including cellular stranding, vacuolation, mucosal erosions, and hyperplasia as previously described (9).

### Toxin A and toxin B ELISA

For the detection of TcdA *in vitro*, overnight cultures of *C. difficile* strains were diluted in TY medium to a starting OD_600_ of 0.05 and grown at 37°C. One milliliter of culture was harvested after 8 and 24 h of growth and centrifuged (5,000 rpm at 4°C) for 5 min. Supernatants were collected and kept at −20°C. Cell pellets were washed in PBS 1× and kept at −20°C. For the toxin A ELISA, PCG4.1 polyclonal antibodies (Bio-techne, Minneapolis, MN) were diluted to 4 ng/mL in PBS and coated overnight on a Maxisorp plate to use as capture antibodies. The wells were then blocked with Superblock blocking buffer (Thermo Fisher Scientific, Waltham, MA). Detection antibodies anti-*C. difficile* toxin A coupled to HRP (LS-Bio, Newark, CA) were then added at a 1:10,000 dilution. The plate was developed by the addition of TMB (Thermo Fisher Scientific, Waltham, MA) followed by a 30-min incubation, and the reaction was stopped by the addition of a 0.2M H_2_SO_4_ solution into the wells. The signal from the test was recorded as absorbance at 450 nm.

Toxin B concentrations from cecal contents were measured as previously described by Zarandi et al. (44) and Girinathan et al. (9) with the following adjustments (9, 44). Microtiter plates were coated with 5 µg/mL of the capture antibody (MAB13508, Abnova, Heidelberg, Germany). Plates were washed three times with PBS-Tween 20 and blocked at RT for 2 h with SuperBlock (Thermo Fisher Scientific, Waltham, MA). Cecal contents were diluted with 1× PBS, vortexed, and centrifuged at 17,000 × *g* for 1 min at 4°C. The sample supernatants and toxoid B standard controls (0–32 ng/mL) (BMLG1550050, Enzo Life Sciences, Southold, NY) were tested in triplicate. Plates were incubated with a biotinylated detection antibody (ab252712, Abcam, Cambridge, UK) and Pierce High-Sensitivity Streptavidin-HRP (Thermo Fisher Scientific, Waltham, MA). Plates were washed with PBS-Tween 20 between incubations. TMB substrate (Thermo Fisher Scientific, Waltham, MA) was used for signal detection at 450 nm with a BioTek Synergy H1 plate reader (BioTek Instruments Inc., Winooski, VT). Values were interpolated in GraphPad Prism 9 (GraphPad, San Diego, CA) to calculate toxin B concentrations. Significant differences between treatment groups were evaluated by a non-parametric Mann-Whitney test with differences declared at a *P*-value ≤ 0.05.

### *In vivo* bacterial RNA sequencing

Snap-frozen cecal contents were weighed into lysis tubes with 0.1 and 0.5 mm beads (Zymo Research, Irvine, CA) and 750 µL DNA/RNA Shield to inactivate nucleases. Cells were lysed at 4°C for a total of 30 min on a Vortex Genie2 (Scientific Industries, Bohemia, NY) fitted with a 2 mL tube holder (Qiagen, Germantown, MD). RNA was purified with the ZymoBIOMICS RNA Miniprep kit (R2001, Zymo Research, Irvine, CA). Purified RNA was concentrated with the RNA Clean and Concentrator-5 kit (R1013, Zymo Research, Irvine, CA) with a second DNAse I step. The concentration and integrity of the purified RNA were quantified with the Qubit 3 fluorometer (Thermo Scientific, Waltham, MA) and 2100 Bioanalyzer (Agilent Technologies, Lexington, MA), respectively. Prior to library prep, qPCR was employed to assess DNA contamination with DNA polymerase III-specific primers (F: 5′-CCCAACTCTTCGCTAAGCAC-3′; R: 5′-TCCATCTATTGCAGGGTGGT-3′). The Zymo-Seq RiboFree Total RNA Library Kit (R3003; Zymo Research, Irvine, CA) was used to generate cDNA libraries following the manufacturer’s instructions. The molar concentration of each individual cDNA library was calculated from the Qubit concentration (ng/µL) and bioanalyzer length. Each sample was diluted to 4 nM and equimolarly pooled. Sequencing on the Illumina MiSeq with the MiSeq 150-cycle v3 kit (MS-102-3001; Illumina, San Diego, CA) was used to assess bacterial versus host enrichment of the libraries. Bacterial read proportions calculated from Bowtie 2 and HTSeq analyses were used to estimate individual sample inputs required to obtain a minimum of 1 million reads for each bacterial species within a sample (9). A second 4 nM pool was generated and sequenced on the Illumina NovaSeq 6000 with the NovaSeq 6000 S4 Reagent v1.5 (300 cycles) kit (20028312, Illumina, San Diego, CA) at the Molecular Biology Core Facilities at the Dana-Farber Cancer Institute (Boston, MA). Data processing and enrichment analyses of the transcriptomes were performed as described by Girinathan et al. (9). Briefly, paired-end reads were mapped to a library of the bacterial and mouse genomes combined (*C. difficile*: NZ_CP049958.1, *C. sardiniense*: JAIKTU000000000.1, *Mus musculus*: GCF_000001635.26) using Bowtie 2 using the “--no-mixed” and “--no-discordant” flags (45). Read pairs with mapping quality under 10 were excluded for non-unique alignment. Mapped reads were assigned to gene features using HTSeq, with a matrix of gene-assigned read counts used for DESeq2 analyses as before, including a 10 aligned read average cutoff needed for analysis (46, 47).

## Data Availability

All RNASeq data were deposited in the National Center for Biotechnology Information’s Gene Expression Omnibus repository under accessions GSE253149 (*C. sardiniense* plus *C. difficile*) and GSE252555 (mono-associated wild-type and mutant *C. difficile* samples). These are repositories containing links to raw sequencing reactions as well as directly containing processed data files and resources.

## SUPPLEMENTAL MATERIAL

Figure S1 mbio.01180-26-s0001.eps Mutant strain growth and toxin production relative to WT.

Figure S2 mbio.01180-26-s0002.eps Mutant strain growth relative to WT in supplemented minimal media.

ASM does not own the copyrights to Supplemental Material that may be linked to, or accessed through, an article. The authors have granted ASM a non-exclusive, world-wide license to publish the Supplemental Material files. Please contact the corresponding author directly for reuse.
